# Pretreatment of Palm Kernel Cake by Enzyme-Bacteria and Its Effects on Growth Performance in Broilers

**DOI:** 10.3390/ani15020116

**Published:** 2025-01-07

**Authors:** Yue Liu, Ying Liu, Yunhe Cao, Chunlin Wang

**Affiliations:** 1 State Key Laboratory of Animal Nutrition and Feeding, College of Animal Science and Technology, China Agricultural University, Beijing 100193, China; ly943959080@163.com (Y.L.); ly13360@163.com (Y.L.); caoyh@cau.edu.cn (Y.C.); 2 Sanya Institute of China Agricultural University, Sanya 572025, China

**Keywords:** palm kernel cake, pretreatment, enzyme-bacteria, solid state fermentation, broilers, growth performance

## Abstract

This study aimed to improve palm kernel cake by reducing anti-nutritional factors with enzymes and enhancing its nutritional value through microbial fermentation. It also examined the effects of these treatments on palm kernel cake in broiler chicken diets. Palm kernel cake was hydrolyzed using xylanase and mannanase under various conditions. Co-fermentation with *Lactobacillus plantarum* QZSL and *Saccharomyces boulardii* mafic-1701 was assessed under different parameters. In the animal experiment, 350 male Cobb broiler chicks were divided into seven groups: a control group provided a corn-soybean meal diet, and groups provided diets containing 10% and 20% palm kernel cake, enzyme-hydrolyzed palm kernel cake, and bacteria-enzyme co-fermented palm kernel cake. Optimal conditions for enzymatic hydrolysis of palm kernel cake are 55 °C, pH 3.0, and a 1:2.5 feed-to-water ratio. Adding 0.1 g xylanase and 1.0 g mannanase to 10 g palm kernel cake for 12 h increased reduced sugar content to 139.33 mg/g and reduced neutral detergent fiber to 43.92%. For solid-state fermentation with *Lactobacillus plantarum* QZSL and *Saccharomyces boulardii* mafic-1701, optimal conditions are 37 °C, 5% inoculation, 20% moisture, 3 days fermentation, and a 7:3 bacterial ratio. Animal experiments showed significant improvements in broilers’ growth, nutrient digestibility, antioxidant capacity, immune function, and intestinal health. Enzyme-bacteria co-fermentation of palm kernel cake boosts its nutritional value and enhances broiler intestinal health.

## Simple Summary

This study focused on improving the nutritional value of palm kernel cake, a by-product of palm oil production, which is often used in animal feed but contains anti-nutritional factors. We used enzymes to break down these factors and then fermented the cake with beneficial microbes. Our experiments showed that these treatments significantly enhanced the nutritional content of the palm kernel cake. We tested this improved feed on broiler chickens and found that it led to better growth, higher feed intake, and improved nutrient absorption. The treated feed also boosted the chickens’ immune systems and overall health. These findings suggest that the reported method made the palm kernel cake a more valuable and effective component of animal feed, potentially reducing feed costs and improving animal welfare. This research is important for the agricultural industry as it offers a sustainable way to enhance animal nutrition and health.

## 1. Introduction

Palm kernel cake (PKC), a byproduct of oil palm processing, is widely produced in tropical countries, with a global output of approximately 58.80 million tons currently [1,2]. It ranks as the fifth largest protein meal feed after soybean meal, rapeseed meal, sunflower meal, and cottonseed meal [3]. PKC, obtained mainly through pressing, has a high ether extract (EE) content ranging from 8.0% and 12.6% [4,5,6,7,8,9]. Its crude protein content is generally between 16% and 20%, offering good solubility, emulsification, and thermal stability, making it a valuable plant protein source [10,11]. Additionally, PKC is cost-effective and non-toxic, and countries with advanced livestock industries, such as the European Union and New Zealand, use it to replace corn and soybean meal to reduce production costs [12].

Research indicates that PKC has a higher bulk density and lower water-holding capacity than other ingredients, allowing it to pass quickly through the poultry digestive tract and potentially enhancing feed intake, which is closely related to animal performance [13]. Its main component, β-mannan, has prebiotic properties that inhibit pathogenic bacteria and boost immunity [14]. However, due to the high proportion of palm shells and peels, PKC has a high crude fiber content, typically between 12.1% and 23.0%. The primary anti-nutritional factors in PKC are non-starch polysaccharides (NSP) [15], which constitute 60% to 74.3% of its content, with β-mannan making up 78%, xyloglucan 6%, and cellulose 12%, reducing its digestibility and limiting its use [16]. Enzymatic hydrolysis and fermentation can reduce these anti-nutritional factors and enhance PKC’s nutritional value. The combined process of enzymatic hydrolysis and microbial fermentation not only leverages the benefits of both methods but also utilizes microorganisms to produce probiotics, organic acids, and natural antimicrobial substances. These processes degrade macromolecules in the feed into smaller molecules, improving nutrient digestion and absorption, and thereby increasing feed utilization efficiency [17,18].

This study employs a gentle, purely biological treatment method, innovatively using a two-step enzyme-microbe synergistic approach to process PKC. Initially, mixed enzymatic hydrolysis with xylanase and mannanase is used to break down macromolecular nutrients, utilizing a reactor for large-scale preparation of enzymatically hydrolyzed PKC. This is followed by optimizing the solid-state fermentation (SSF) process with laboratory-isolated strains of *Saccharomyces boulardii* mafic-1701 (*S. boulardii* mafic-1701) and *Lactobacillus plantarum* QZSL (*L. plantarum* QZSL) to further enhance the nutritional value of the hydrolyzed PKC. Finally, the effects of pretreated PKC on broiler growth performance, serum indices, and gut microbiota are comprehensively analyzed. Through these approaches, we explore the feasibility of large-scale application of PKC in animal production, providing new perspectives for the efficient utilization of agricultural byproducts.

## 2. Materials and Methods

### 2.1. Optimization of Mixed Enzymatic Hydrolysis Conditions of Palm Kernel Cake

#### 2.1.1. Enzyme Production and Activity Measurement

PKC was provided by Guangdong Junyou Feed Company Xylanase was produced in the Feed Industry Center of the Ministry of Agriculture and Rural Affairs (MAFIC) at China Agricultural University, achieving an activity of 5755.63 U/mL after 126 h of methanol induction in 30 L fermenter. Mannanase was provided by Shandong Longcote Enzyme Preparation Company (Linyi, China), with an activity of 20,000 U/g. Enzyme activities were measured using the DNS method, with xylanase activity defined as the amount of enzyme required to produce 1 μmol of xylose per minute under specific conditions (55 °C, pH 3.0). The activity of β-mannanase was determined according to the national standard GB/T 36861-2018 [19]. Reducing sugar (RS) content was measured by extracting the sugar from the sample, diluting it, and using a spectrophotometer at 540 nm.

#### 2.1.2. Chemical Composition and Content Determination

The determination of crude ash, ether extract (EE), crude protein (CP), neutral detergent fiber (NDF), and acid detergent fiber (ADF) in PKC was carried out according to the standards in China: GB/T 20806-2006 [20], GB/T 6432-1994 [21], GB/T 6435-2006 [22], GB/T 20806-2006 [20], and NY/T 1459-2007 [23]. The amino acid content in PKC was determined according to previously reported methods [24,25].

#### 2.1.3. Enzymatic Hydrolysis Experiments

A 10 g sample of PKC (passed through a 40-mesh sieve) was placed into an Erlenmeyer flask. For testing the single enzyme addition, different amounts of each of xylanase and mannanase (0.05, 0.1, 0.5, 1.0, and 1.5 g) were added and pretreated at 50 °C and 180 rpm for 12 h. For testing the dual enzyme addition, various combinations of xylanase and mannanase concentrations were tested. For testing the feed-to-water ratio, 0.1 g of xylanase and 1.0 g of mannanase were added and pretreated at 55 °C and 180 rpm. For testing the hydrolysis time, pretreatment times of 4, 8, 12, and 16 h were tested at 55 °C and 180 rpm. For testing pH and temperature, the following levels were used and included buffer solutions with pH values of 3.0, 3.5, 4.0, 4.5, and 5.0 and temperatures of 40, 45, 50, 55, and 60 °C, respectively. After all experiments, samples were dried at 65 °C, equilibrated at room temperature for 24 h, and the RS and neutral detergent fiber content were determined.

### 2.2. Co-Fermentation of Palm Kernel Cake by Lactobacillus plantarum QZSL and Saccharomyces boulardii Mafic-1701

#### 2.2.1. Microbial Strains and Analytical Methods

*L. plantarum* QZSL was provided by the Department of Nutrition and Health, China Agricultural University. *Escherichia coli* (*E. coli*) K88, K99, and *Staphylococcus aureus* CVCC were preserved strains from MAFIC. Total acid content was measured according to the GB 12456-2021 standard [26]. For viable cell count, 3 g of fermented sample was mixed with 27 mL of physiological saline to prepare a bacterial suspension, which was then gradient diluted and plated. After 24 h of incubation, colonies were counted. For pH measurement, 1 g of fermented sample was mixed with 9 mL of distilled water, and the pH value was measured. The growth curve of *L. plantarum* QZSL was determined by inoculating 1% into MRS medium, incubating at 37 °C for 24 h, and monitoring absorbance and pH every 3 h. The growth curve of *S. boulardii* mafic-1701 was determined by inoculating 1% into YPD medium, incubating at 37 °C for 40 h, and monitoring absorbance every 2 h.

#### 2.2.2. Solid-State Fermentation Procedure and Formulation

(1) A 2.5 kg sample of PKC and 1.35 L of water was sterilized at 121 °C for 15 min, cooled, then 25 g of xylanase and 25 g of mannanase was added, mixed well, and pretreated at 55 °C for 12 h.

(2) A 0.5 kg sample of wheat bran and 100 g of brown sugar was dissolved in 100 mL of water, sterilized at 115 °C for 15 min, cooled, and mixed well.

(3) The mixture from step 2 was added to the PKC from step 1 and the bacterial solution was added, mixed well, and distributed into anaerobic fermentation bags, with 1.0 kg per bag and 5 replicates per level. The blank control group had no bacteria and enzymes added. The fermentation bags were sealed and incubated at 37 °C for 5 days.

The experiment on the co-fermentation conditions of PKC by *L. plantarum* QZSL and *S. boulardii* mafic-1701 was conducted according to the SSF steps and the formulations shown in Table 1 and Table 2.

### 2.3. Impacts of Palm Kernel Cake on Broiler Health and Performance

The enzymatic hydrolysis product used in this experiment is a mixture of xylanase and mannanase hydrolyzed palm kernel cake. It was produced in large quantities using a 30 L reaction kettle and dried at low temperature in an oven to obtain Enzymatic Hydrolysis of Palm Kernel Cake (EPKC). The enzymatically hydrolyzed product was then subjected to co-fermentation with *L. plantarum* QZSL and *S. boulardii* mafic-1701 in an anaerobic breathing bag according to the bacterial enzyme co-fermentation formula. After natural air drying, Palm Kernel Cake fermented with bacteria and enzyme (FEPKC) was obtained. A total of 350 one-day-old Cobb broiler male chicks were randomly divided into 7 treatments, with 5 replicates per treatment and 10 chicks per replicate. The nutritional composition and levels of the diets are shown in Table 3 and the formulation of the feed followed the standards of GB/T 5916-2020 [27]. The experiment lasted for 42 days and was divided into two phases: the early phase (1–21 days) and the late phase (22–42 days). The experimental design is as follows: Control Group: fed a basal corn-soybean meal diet. PKC1 and PKC2: corn replaced with 10% and 20% PKC, respectively; EPKC1 and EPKC2: corn replaced with 10% and 20% enzymatically hydrolyzed PKC, respectively; and FEPKC1 and FEPKC2: corn replaced with 10% and 20% fungal-enzyme co-fermented PKC, respectively.

This experiment was conducted in the metabolism room of the MAFIC at China Agricultural University. Before the experiment, all necessary equipment such as chicken coops, water troughs, and feed troughs were thoroughly disinfected to prepare for the arrival of the chicks. Throughout the experiment, the broilers had free access to feed and water. Initially, tower-type snap-on poultry drinkers were used, and as the broilers grew larger, nipple-type automatic drinkers were employed. The broilers were provided with 24 h lighting daily, and temperature, humidity, and ventilation conditions were strictly controlled according to broiler management standards. The experimental diet was fed three times a day, and daily records were kept of temperature, humidity, mortality, and broiler growth. Any abnormal phenomena were closely observed. Weekly feed consumption was recorded. Each broiler was wing-tagged and weighed individually at the start of the experiment, and fasting weights were taken at 21 days and 42 days, with feed withheld for 12 h before weighing to ensure continuous fresh water supply. In this experiment, the FEPKC was prepared according to the method described above. On the 21st and 42nd days of the experiment, samples were collected. Growth performance indicators were measured for all broilers, while other indicators were assessed by collecting one experimental chicken with a body weight close to the average from each replicate group and fecal samples for the determination of apparent nutrient digestibility were collected on a replicate basis, totaling 35 samples. Serum sample collection: chickens were fasted for 12 h before blood collection. Blood was collected from the jugular vein (10 mL) and left at room temperature for 30 min. The samples were then centrifuged at 3000 r/min for 10 min to separate the serum, which was transferred to centrifuge tubes and stored at −20 °C for subsequent biochemical analysis. Tissue sample collection: the thymus, spleen, and bursa of Fabricius were separated from the experimental chickens, and excess fat was removed before weighing to calculate the immune organ index. Growth performance: all broilers were weighed, and feed intake was recorded. Average daily gain (ADG), average daily feed intake (ADFI), and feed-to-gain ratio (F:G) were calculated for the early phase (days 0–21), late phase (days 22–42), and the entire period (days 1–42). Nutrient digestibility: to evaluate nutrient digestibility, 0.3% chromium trioxide was added to the diet as an external marker. For each treatment, 500 g of feed samples were collected and stored in a −20 °C freezer. Fecal samples were collected from days 19 to 21 and days 40 to 42, and stored at −20 °C to determine nutrient digestibility. Feed and fecal samples were freeze-dried for 72 h and then ground to pass through a 40-mesh sieve. The chromium (Cr) content in the feed and fecal samples was determined using an atomic absorption spectrophotometer (Hitachi Z-5000, Tokyo, Japan) according to the national standard of the People’s Republic of China, GB/T 13088-2006 [28]. The apparent total tract digestibility (ATTD) of each nutrient was calculated using the following formula:

Apparent digestibility of dietary nutrients (%) = 1 − (Cr content in diet/Cr content in feces) × (Nutrient content in feces/Nutrient content in diet) × 100%

Serum immune indicators and antioxidant capacity were measured using enzyme-linked immunosorbent assay (ELISA) kits. The fat surrounding the spleen, thymus, and bursa of Fabricius was carefully removed before weighing. The immune organ index of broilers was calculated using the following formula:

Immune organ index (%) = (Weight of immune organ/Live weight) × 100

Jejunum tissue samples were fixed for 48 h, dehydrated, and embedded in paraffin. Sections were cut using a microtome, deparaffinized, and stained with hematoxylin and eosin. The morphology of the jejunum was observed under a microscope equipped with an integrated digital imaging analysis system. At least six intact and clear villi were selected from each intestinal sample to measure crypt depth and villus height. The gut microbiota of broilers was analyzed through 16S rRNA sequencing conducted by Majorbio Bio-Pharm Technology Co., Ltd. (Shanghai, China). DNA was extracted from cecal chyme using a fecal DNA extraction kit (Omega Bio-tek, Norcross, GA, USA). DNA quality was assessed by 1% agarose gel electrophoresis, and DNA concentration and purity were measured using a NanoDrop spectrophotometer (Thermo Scientific, Wilmington, DE, USA). PCR amplification was performed using bacterial universal primers 338F (5′-barcode ACTCCTACGGGAGGCAGCAG-3′) and 806R (5′-GGACTACHVGGGTWTCTAAT-3′). The PCR products were then subjected to quality control, recovery, and library construction. Sequencing was performed using the Illumina HiSeq platform (Illumina, San Diego, CA, USA). Microbial diversity analysis was conducted using the I-Sanger cloud platform (https://www.majorbio.com/web/www/index, accessed on 13 January 2023).

### 2.4. Statistical Analysis

Data were statistically analyzed using SPSS (2.0, Chicago, IL, USA). Growth performance and apparent nutrient digestibility were analyzed with replicates as the statistical unit, while serum indicators and intestinal morphology were analyzed with each broiler as the statistical unit. ANOVA was performed for variance analysis, and multiple comparisons were conducted using the Bonferroni and Tukey method. Statistical results were considered extremely significant at *p* < 0.01 and significant at *p* < 0.05. Microbial diversity analysis was performed using the I-Sanger cloud platform from Shanghai Majorbio Bio-Pharm Technology Co., Ltd. The raw reads were deposited into the National Center for Biotechnology Information Sequence Read Archive (SRA) database. SRA number is PRJNA1184972.

## 3. Results

### 3.1. Optimization of Enzymatic Hydrolysis Conditions for Palm Meal Mixture

The nutritional composition of PKC is shown in Table 4.

**Figure 1:**
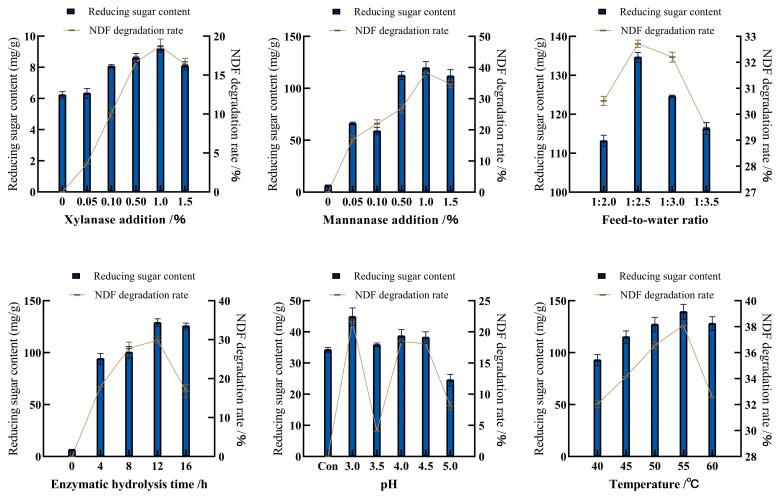
Effects of enzymatic hydrolysis conditions on reducing sugar content and neutral detergent fiber degradation rate.

The RS content of untreated PKC is relatively low, at 6.22 mg/g, with an NDF content of 70.97%. The addition of 1.00 g of xylanase or mannanase alone increased the RS content and NDF degradation rate of PKC, with mannanase showing a significant effect (*p* < 0.05). The RS content released by PKC was 119.58 mg/g, which was 18 times higher than that of the control group, and the NDF degradation rate was 38.37% (Figure 1). When the two enzymes were added simultaneously, the combination of 0.1 g xylanase and 1.0 g mannanase had the best effect, where the RS content released by PKC reached 131.98 mg/g which was significantly higher than that of the control group (*p* < 0.05) and a resultant NDF content of 50.09% (Table 5). When the feed-to-water ratio was 1:2.5, the RS content and NDF degradation rate of PKC were the highest. The best effect was achieved when the pretreatment time was 12 h, with no significant difference when the time was further extended. The enzymatic hydrolysis effect was best at pH 3.0. At 55 °C, the RS content and NDF degradation rate of PKC were the highest (Figure 1). Therefore, the optimal treatment conditions were the addition of 0.1 g xylanase and 1.0 g mannanase with a feed-to-water ratio of 1:2.5 and pretreated for 12 h with pH 3.0 at 55 °C. The RS content of PKC treated with xylanase and mannanase simultaneously reached a maximum of 139.33 mg/g, 22 times higher than that of untreated PKC (*p* < 0.05); NDF content reached a minimum of 43.92%, 30.17% lower than that of untreated PKC (*p* < 0.05).

### 3.2. Co-Fermentation of Palm Kernel Cake by Saccharomyces boulardii Mafic-1701 and Lactobacillus plantarum QZSL

Based on Table 6, the optimal ratio of *L. plantarum* QZSL to *S. boulardii* mafic-1701 is 7:3. At this ratio, the crude protein content is significantly higher than other treatment groups (*p* < 0.05), the RS content was significantly higher than other treatment groups (*p* < 0.05), the pH value was significantly lower (*p* < 0.05), and the total acid content was 3.15 g/kg. The viable cell count after fermentation was 5.6 × 10^5^ CFU/mL, significantly higher than the other groups (*p* < 0.05). Based on Table 7, the optimal inoculation amount was 5% where the RS content was significantly higher than for the 10% and 15% inoculation groups (*p* < 0.05), the NDF content was the lowest, and the crude protein content was significantly higher than before fermentation (*p* < 0.05). The viable cell count after fermentation was 1.1 × 10^6^ CFU/mL, significantly higher than the other groups (*p* < 0.05). Based on Table 8, the optimal moisture content was 20% where the RS content was significantly higher than the 28% and 36% moisture content groups (*p* < 0.05), and the NDF content was the lowest. The total acid content was 5.53 g/kg, significantly higher than other treatment groups (*p* < 0.05). The viable cell count after fermentation was 2.0 × 10^6^ CFU/mL, significantly higher than the other groups (*p* < 0.05). Based on Table 9, the optimal fermentation time was 3 days where the RS content was significantly higher than the other groups (*p* < 0.05), and the NDF content was the lowest at 50.64%. The viable cell count after fermentation was 5.4 × 10^6^ CFU/mL, significantly higher than the other groups (*p* < 0.05).

The results indicated that the optimal SSF conditions were at 37 °C with an inoculation amount of 5% and moisture content of 20%, including 3 days of fermentation and a bacterial ratio of *L. plantarum* QZSL to *S. boulardii* mafic-1701 at 7:3.

### 3.3. Impacts of Palm Kernel Cake on Broiler Performance and Health

**Figure 2:**
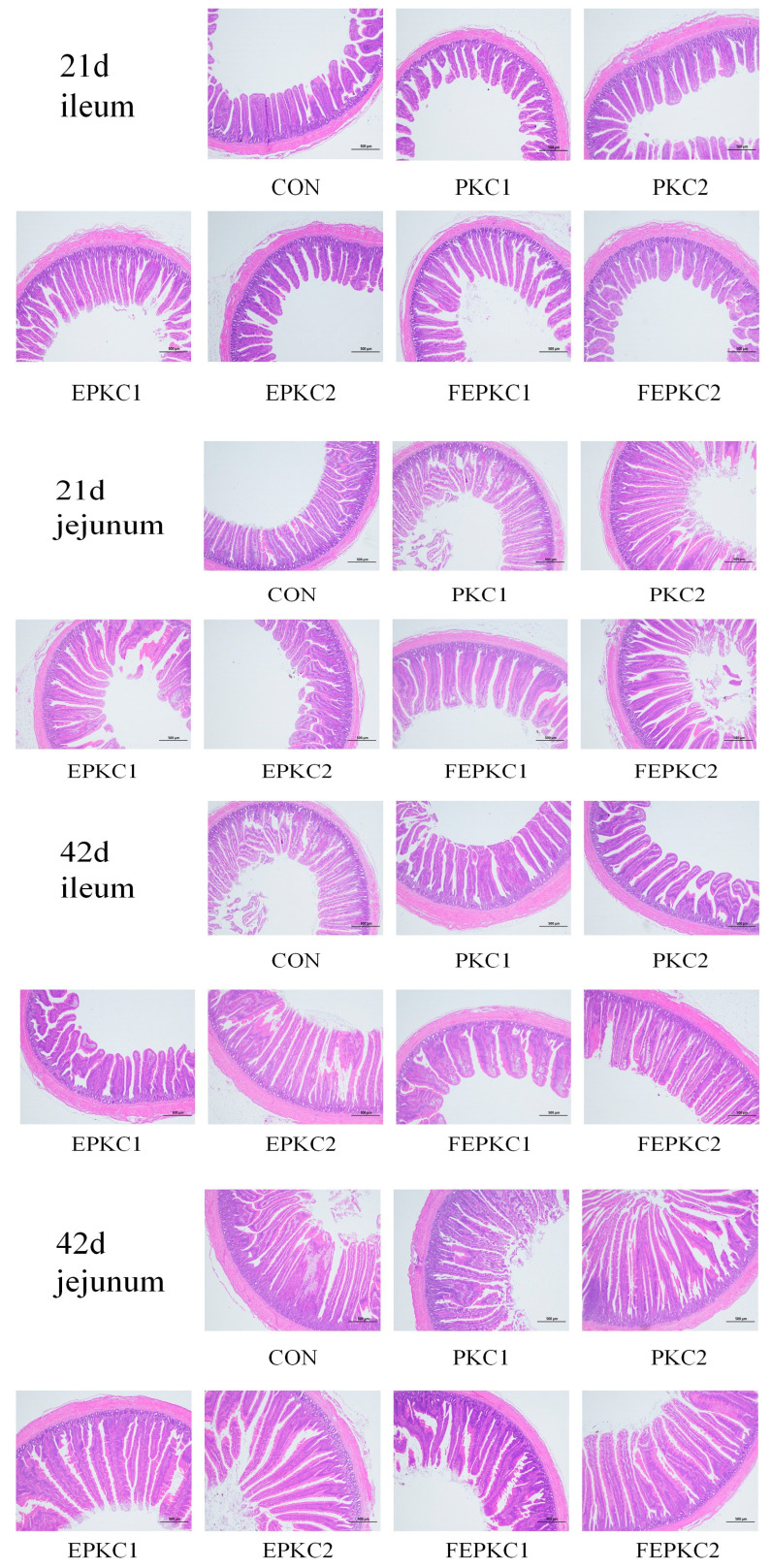
Intestinal morphology of broilers.

As shown in Table 10, during the early stage of the experiment, the average daily weight gain of broilers fed the FEPKC1 diet was significantly higher than that of the other treatment groups (*p* < 0.05). In the later stage of the experiment, the average daily weight gain of the FEPKC1 group was significantly higher than that of the control group, PKC group, and EPKC group (*p* < 0.05). Over the entire experiment, feed intake of the PKC treatment groups was higher than that of the control group. Replacing corn with the FEPKC1 diet significantly increased the average daily weight gain of broilers (*p* < 0.05). The DM and CP digestibility of the FEPKC1 diet was significantly higher than that of the other treatment groups in the early stage of the experiment (*p* < 0.05), and the EE digestibility in the later stage was also significantly higher compared to the control group (*p* < 0.05). Overall, replacing corn with the FEPKC1 diet improved ADF digestibility (Table 11). In terms of serum indicators, all treatment groups had significantly increased T-SOD levels and decreased MDA levels (*p* < 0.05), but there was no significant effect on T-AOC (Table 12). The FEPKC2 group had significantly increased IgA content in the early stage of the experiment (*p* < 0.05), and all treatment groups significantly increased IgA content and significantly decreased IgM levels in the later stage (*p* < 0.05) (Table 13). There were no significant differences in the immune organ index across all treatment groups (*p* > 0.05) (Table 14). The EPKC2, FEPKC1, and FEPKC2 groups had significantly improved the villus height to crypt depth ratio of the jejunum and ileum in the later stage of the experiment (*p* < 0.05) (Table 15; Figure 2).

**Figure 3:**
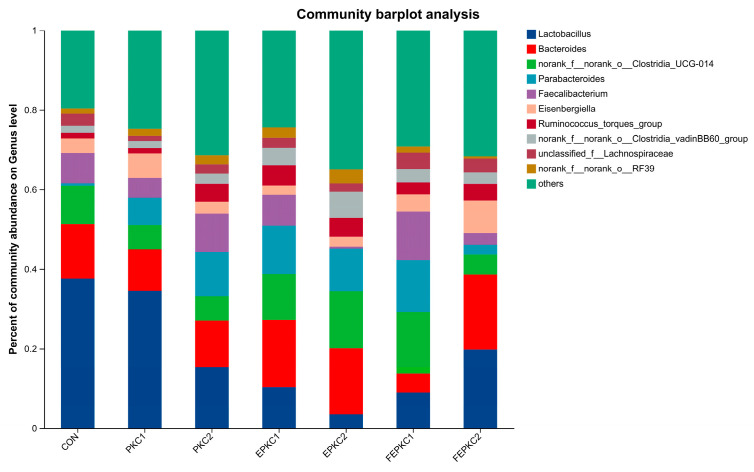
Effects of pretreated PKC on intestinal flora structure in broilers at 21 d.

**Figure 4:**
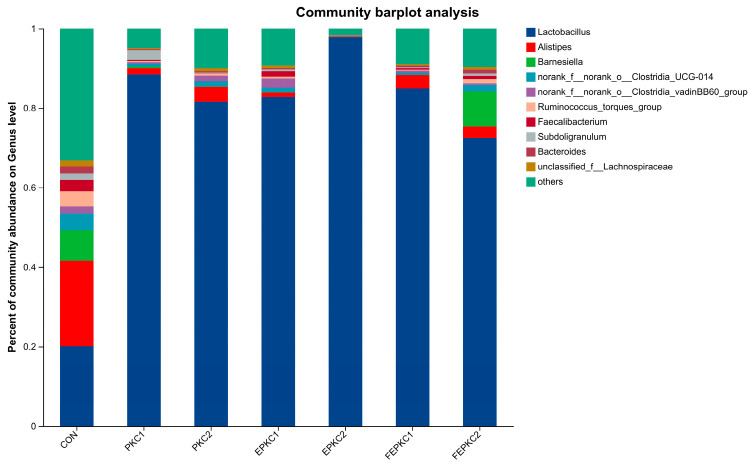
Effects of pretreated PKC on intestinal flora structure in broilers at 42 d.

Combining the results of 21 days (Figure 3) and 42 days (Figure 4), the bacterial species in the intestinal microbiota of broilers among the groups basically remained unchanged. The dominant positions of *Eisenbergiella* and *norank_f_norank_o_RF39* in relative abundance at 42 days were replaced by *Alistipes* and *Subdoligranulum*. However, the structure of the intestinal microbiota of broilers underwent significant changes, especially at 42 days. Compared to the control group, the relative abundance of *Lactobacillus* in the intestinal microbiota of broilers in all treatment groups significantly increased and became dominant representing the greatest relative proportion. Additionally, it is noteworthy that compared to the other treatment groups, FEPKC2 significantly increased the relative abundance of *Barnesiellaceae*.

## 4. Discussion

### 4.1. Optimization of Enzymatic Hydrolysis Conditions for Palm Kernel Cake

PKC contains 78% β-mannan within its NSP [29]. Therefore, researchers have tried various methods to reduce its content. Steam pretreatment and treatment of PKC with mannanase from *Bacillus amyloliquefaciens* were shown to significantly increase protein content and decrease hemicellulose and lignin content, releasing a large amount of RS [30]. Adding ammonium sulfate fermentation to improve the quality of PKC, and subsequent enzymatic hydrolysis with a complex enzyme system including xylanase, cellulase, and mannanase increased the digestibility of protein and crude fiber (*p* < 0.05) [31]. This experiment attempts to use a single biological treatment method, namely the mixed enzymatic hydrolysis of xylanase and mannanase, as the high β-mannan content makes mannanase more effective than xylanase when used alone. Increasing the dosage of either enzyme alone raised RS content and NDF degradation rate, but effectiveness declined beyond 1.0 g, likely due to enzyme binding site saturation and potential self-degradation. The combination of 0.1 g xylanase and 1.0 g mannanase showed superior results, possibly due to synergistic effects where xylanase disrupted cell walls and released mannan with enhancing mannanase hydrolysis efficiency [32]. This significantly reduced NSP’s anti-nutritional effects and was consistent with previous studies using enzyme mixtures [33]. The optimal pH for xylanase and mannanase was 3.0–5.0, making acidic conditions favorable for PKC hydrolysis. PKC’s gritty and fibrous texture caused rapid water absorption and a viscous paste at feed-to-water ratios below 1:2.5, hindering enzyme contact and reducing degradation efficiency. A ratio of 1:3 lowered substrate concentration and did not maximize enzymatic capacity. Despite good thermal stability, enzyme effectiveness decreased over 50 °C, likely due to water evaporation altering the feed-to-water ratio. Under optimal conditions, xylanase and mannanase treatment significantly increased RS content, produced prebiotic manno-oligosaccharides, and reduced NDF content (*p* < 0.05), which could enhance the nutritional value and economic benefits of PKC.

### 4.2. Solid-State Fermentation of Palm Kernel Cake with Lactobacillus plantarum QZSL and Saccharomyces boulardii Mafic-1701

The use of combined strains for SSF has been proven to be beneficial for various raw materials. The combination of *L. plantarum* QZSL and *S. boulardii* mafic-1701 was used to enhance the nutritional value of an African fermented cassava food [34]. *Saccharomyces cerevisiae* and *L. plantarum* were used to improve the nutritive and antioxidant properties of hull-less barley [35]; in addition, *L. plantarum* and *Rhizopus oryzae* were used for whole oat [36]; *L. plantarum* and *Aspergillus niger* for whole wheat bran [37]; *Bacillus velezensis* and *L. plantarum* for soybean meal [38]; and *Saccharomyces cerevisiae* and *L. plantarum* for whole wheat bran bread [39]. Additionally, *L. plantarum* and *Lactobacillus brevis* efficiently produced lactic acid from cellulose and xylan in bagasse [40]. Our previous studies showed that the improvement in feed conversion ratio and reduction in diarrhea rate in weaned piglets provided diets supplemented with *S. boulardii* mafic-1701 may be associated with enhanced antioxidant activity, anti-inflammatory responses, and improved intestinal microbial ecology [34]. Therefore, following enzymatic pretreatment to reduce anti-nutritional factors, this study attempts to combine the benefits of enzymatic hydrolysis and SSF with *L. plantarum* QZSL and *S. boulardii* mafic-1701. During fermentation, *S. boulardii* mafic-1701 consumed oxygen, creating anaerobic conditions favorable for *L. plantarum* QZSL growth. This process degraded NSPs in PKC into monosaccharides, such as manno-oligosaccharides, which acted as prebiotics to promote probiotic proliferation [41]. As oxygen is depleted, *L. plantarum* QZSL rapidly grows, producing organic acids like lactic acid, lowering pH, imparting a sour aroma to the feed, inhibiting harmful microorganisms, and improving feed palatability and storage [42]. Co-fermentation with *L. plantarum* QZSL and *S. boulardii* mafic-1701 increased crude protein content, which was consistent with other studies on fermented feed ingredients. The increase in crude protein may result from microbial consumption of organic matter, leading to a “concentration effect”, though fermentation may also cause the loss of vitamins and amino acids [43]. The primary goal of SSF is to enhance the nutritional value and palatability of PKC, with β-mannan being the main anti-nutritional factor. Therefore, the key factor in optimizing fermentation conditions is the release of RS. When the ratio of *L. plantarum* QZSL and *S. boulardii* mafic-1701 was 7:3, the release of RS was significantly higher than in the other treatments, indicating effective β-mannan degradation. This ratio also resulted in the highest crude protein content, total acid content, and viable cell count, supporting the conclusion that 7:3 was the optimal bacterial ratio. The fermented PKC became porous and aromatic, enhancing palatability and extending storage time. The optimal temperature for SSF was 37 °C, ensuring normal microbial growth and metabolism. The moisture content of the medium was crucial; low moisture reduced nutrient and metabolite diffusion, affecting enzyme activity and microbial growth, while excessive moisture increased the risk of mycotoxin contamination [44]. The optimal moisture level was 20% with the highest RS release, lowest NDF content, and viable cell count of 2.0 × 10^6^ CFU/mL achieved. Insufficient fermentation time resulted in poor fermentation, while excessive time led to nutrient depletion and microbial autophagy. A fermentation time of 3 days yielded the highest RS content and viable cell count, thus balancing production efficiency and cost [45].

### 4.3. Effects of Palm Kernel Cake on Broiler Performance, Serum Indices, and Intestinal Health

The crude fiber content of PKC is acceptable for most ruminants but is not for poultry. Studies showed that SSF can biologically degrade PKC, improving its nutritional quality and serving as an alternative ingredient to enhance the health and growth performance of broilers [46,47,48,49,50]. However, there is contrary evidence: feeding high-fat fermented PKC improved gut bacterial populations and blood lipid concentrations but did not enhance broiler growth performance [51]. Feeding *Lactobacillus*-fermented PKC to broiler chickens improved intestinal development and the expression of glucose and amino acid transporters but had no effects on nutrient digestibility and digestive enzyme activity [52]. In this study, the inclusion levels of PKC were primarily balanced between the nutritional requirements of broilers and economic benefits, while also attempting to enhance the existing safe inclusion levels [1]. This study showed that FEPKC instead of 10% corn significantly increased ADFI and ADG of broilers, demonstrating its potential to improve broiler performance, while untreated PKC reduced performance.

Broilers fed PKC diets had higher ADFI than the control group, which compensated for the lower energy content of PKC. Enzyme-bacteria co-fermentation reduced anti-nutritional factors and enhanced the nutritional properties of PKC, aligning with the observed improvements in growth performance. In the early phase, the DM digestibility of the FEPKC1 group was significantly higher than the control. In the later phase, the ADF digestibility of the FEPKC1 group was significantly improved, indicating enhanced nutrient digestibility with 10% FEPKC substitution. This contrasts with previous findings, where 15% fermented PKC did not significantly improve, possibly due to differences in fiber content and gut microbiota structure [53,54]. T-SOD, an antioxidant enzyme, maintains oxidative balance in animals, while MDA reflects lipid peroxidation levels. All PKC diet groups significantly increased T-SOD levels and decreased MDA levels, indicating enhanced antioxidant capacity. This is consistent with findings that manno-oligosaccharides enhanced serum SOD activity and reduced MDA levels, improving antioxidant capacity [55,56]. FEPKC2 significantly increased serum IgA levels, likely due to probiotic activation of mucosal protection mechanisms and enhanced immune function. Intestinal morphology indicators, such as villus height, crypt depth, and villus-to-crypt ratio, reflect intestinal permeability, absorption, and mucosal function. Higher villus height indicates greater nutrient absorption capacity, while deeper crypts suggest faster tissue metabolism and lower production efficiency. In this study, PKC diets did not significantly affect villus height and crypt depth, but EPKC2, FEPKC1, and FEPKC2 significantly improved the jejunal villus-to-crypt ratio, and PKC2, EPKC1, and FEPKC2 improved the ileal ratio, suggesting higher intestinal absorption and development. The gut microbiota of broilers plays a crucial role in digestive capacity, immunity, and overall health. In terms of gut microbiota structure, there were no significant differences at 21 days when broilers were fed diets containing the alternative feed, PKC. However, at 42 days, all treatment groups formed a community structure dominated by the probiotic *Lactobacillus*, significantly improving the general profile of the gut microbiota. As a beneficial bacterium, *Lactobacillus* helped maintain gut microbial balance, improved growth performance, promoted digestion and absorption, and enhanced immunity [57]. Additionally, at 42 days, the appearance of *Subdoligranulum* in the gut microbiota, another beneficial bacterium is an important producer of short-chain fatty acids with anti-inflammatory effects [57]. *Barnesiella*, a characteristic bacterium in the FEPKC2 group, is capable of fermenting carbohydrates to produce volatile fatty acids and degrading various glycosidic bonds, including oligosaccharides and polysaccharides [58]. This indicates that the FEPKC2 group contains more fermentable oligosaccharides, which may be one of the reasons for promoting broiler growth. In conclusion, the above results suggested that the simultaneous treatment of palm kernel cake with enzymes and bacteria can improve the cecal microecological environment of broilers at 42 d. From an economic cost perspective, the average price per ton of palm kernel cake is approximately half that of corn, and the processing process clearly yields benefits greater than the costs. Since this experiment entirely used biological methods for pretreatment, the processing and additive costs in large-scale production are relatively low, and some valuable by-products are generated during fermentation. Importantly, using biological methods for pretreatment has a smaller environmental impact compared to traditional chemical methods, and this environmental benefit is the cornerstone for the long-term development of the industry.

## 5. Conclusions

The optimal treatment conditions for enzymatic hydrolysis of PKC are 55 °C, pH 3.0, and a feed-to-water ratio of 1:2.5. Under these conditions, adding 0.1 g of xylanase and 1.0 g of mannanase to 10 g of PKC and hydrolyzing for 12 h resulted in an RS content of 139.33 mg/g and a neutral detergent fiber degradation rate of 38.11%. The optimal treatment conditions for SSF with *Lactobacillus plantarum* QZSL and *Saccharomyces boulardii* mafic-1701 were determined to be at 37 °C, 5% inoculation, 20% moisture, and 7:3 bacterial ratios with a fermentation duration of 3 days. Inclusion in broiler diets with differently treated PKC showed that enzyme-bacteria co-fermented PKC significantly increased average daily feed intake and average daily gain, enhanced nutrient digestibility and immune function, optimized gut microbiota structure, and promoted intestinal health. These findings suggested that enzyme-bacteria co-fermented PKC can effectively improve the growth performance and overall health of broilers.

## Figures and Tables

**Table 1:** Basic formula of culture medium.

Ingredients	PKC	Wheat Bran	Brown Sugar	Water	Xylanase	Mannanase	*L. plantarum* QZSL	*S. boulardii* mafic-1701	Total
Formulations (kg)	2.5	0.5	0.1	1.35	0.025	0.025	0.25	0.25	5
Proportions (%)	50	10	2	27	0.5	0.5	5	5	100

**Table 2:** Optimization of fermentation conditions.

	Treatment Groups	*L. plantarum* QZSL (mL)	*S. boulardii* Mafic-1701 (mL)
Bacterial ratios	T1 (5:5)	250	250
	T2 (7:3)	350	150
	T3 (3:7)	150	350
Inoculation amount	T1 (5%)	166	71
	T2 (10%)	350	150
	T3 (15%)	525	225
Water content	T1 (20%)	166	71
	T2 (28%)	166	71
	T3 (36%)	166	71
Fermentation time, d	T1 (3 d)	166	71
	T2 (5 d)	166	71
	T3 (7 d)	166	71

Note: The content of wheat, bran, brown sugar, water, xylanase, and mannanase is consistent with the basic formula.

**Table 3:** Composition and nutrient levels of the diet from 1 to 42 days (%, as-fed basis).

Items	1 to 21 Days							22 to 42 Days						
	CON	PKC1	PKC2	EPKC1	EPKC2	FEPKC1	FEPKC2	CON	PKC1	PKC2	EPKC1	EPKC2	FEPKC1	FEPKC2
Corn	53.16	47.84	42.53	47.84	42.53	47.84	42.53	62.57	56.31	50.06	56.31	50.06	56.31	50.06
PKC	0.00	5.32	10.63	5.32	10.63	5.32	10.63	0.00	6.26	12.51	6.26	12.51	6.26	12.51
Soybean meal	35.38	32.77	30.97	32.72	30.86	33.01	32.94	24.75	24.36	20.37	24.36	20.37	25.36	20.65
Soybean oil	3.10	4.00	5.00	4.00	5.00	4.77	5.00	3.80	5.64	6.10	5.64	6.10	5.43	5.86
Corn gluten powder	3.50	5.00	6.00	5.00	6.00	4.28	4.23	4.50	2.98	7.00	2.98	7.00	2.66	6.61
Calcium dihydrogen phosphate	1.65	1.88	1.67	1.95	1.87	1.71	1.48	1.54	1.56	1.19	1.56	1.19	1.27	1.51
Limestone	1.36	1.30	1.29	1.28	1.19	1.24	1.29	1.15	0.88	1.04	0.88	1.04	1.00	0.90
Salt	0.30	0.30	0.30	0.30	0.30	0.30	0.30	0.30	0.30	0.30	0.30	0.30	0.30	0.30
*L*-Lysine hydrochloride	0.31	0.36	0.39	0.36	0.40	0.29	0.35	0.28	0.35	0.35	0.35	0.35	0.27	0.38
*DL*-Methionine	0.15	0.14	0.14	0.14	0.14	0.20	0.15	0.10	0.17	0.07	0.17	0.07	0.13	0.09
*L*-Threonine	0.12	0.11	0.12	0.11	0.12	0.14	0.12	0.04	0.12	0.03	0.12	0.03	0.04	0.10
*L*-Tryptophan	0.00	0.00	0.00	0.00	0.00	0.00	0.00	0.00	0.09	0.01	0.09	0.01	0.00	0.03
Chromium oxide	0.25	0.25	0.25	0.25	0.25	0.25	0.25	0.25	0.25	0.25	0.25	0.25	0.25	0.25
Phytase	0.02	0.02	0.02	0.02	0.02	0.02	0.02	0.02	0.02	0.02	0.02	0.02	0.02	0.02
Choline chloride	0.20	0.20	0.20	0.20	0.20	0.20	0.20	0.20	0.20	0.20	0.20	0.20	0.20	0.20
Vitamin-mineral premix ^1^	0.50	0.50	0.50	0.50	0.50	0.50	0.50	0.50	0.50	0.50	0.50	0.50	0.50	0.50
Nutrient content														
Metabolizable energy, Mcal/kg	2.98	2.98	2.98	2.98	2.98	2.98	2.98	3.15	3.15	3.15	3.15	3.15	3.15	3.15
Crude protein	22.81	23.00	23.04	22.98	23.09	22.84	22.99	19.40	19.33	19.55	19.33	19.56	19.30	19.58
Calcium	0.99	1.03	0.98	1.04	0.99	0.98	0.95	0.78	0.78	0.76	0.78	0.76	0.76	0.78
Available phosphorus	0.45	0.51	0.48	0.52	0.52	0.48	0.45	0.41	0.43	0.43	0.43	0.43	0.43	0.44
Lys	1.22	1.22	1.22	1.22	1.22	1.22	1.22	0.98	1.02	0.97	1.02	0.97	0.98	0.99
Met	0.49	0.49	0.49	0.49	0.49	0.51	0.50	0.41	0.46	0.40	0.46	0.40	0.42	0.42
Met + Cys	0.92	0.91	0.91	0.91	0.91	0.91	0.91	0.78	0.81	0.76	0.81	0.76	0.78	0.78
Thr	0.84	0.83	0.83	0.83	0.83	0.83	0.83	0.64	0.70	0.63	0.70	0.63	0.63	0.70
Trp	0.22	0.21	0.21	0.21	0.21	0.22	0.22	0.17	0.25	0.17	0.25	0.17	0.17	0.19

Note: ^1^ Premix provided the following per kg of complete diet: Vitamin A, 12,000 IU; Vitamin D_3_, 2500 IU; Vitamin E, 30 IU; Vitamin K_3_, 3.0 mg; Vitamin B_1_, 1.0 mg; Vitamin B_2_, 5.2 mg; Vitamin B_6_, 2.0 mg; Vitamin B_12_, 12.0 g; nicotinic acid, 40.0 mg; pantothenic acid, 15.0 mg; folic acid, 0.4 mg; biotin, 0.04 mg; choline chloride, 400.0 mg; Fe, 90.0 mg; Cu, 10.0 mg; Zn, 80.0 mg; Mn, 16.0 mg; I, 0.24 mg; Se, 0.3 mg.

**Table 4:** Nutritional components of PKC (%, DM).

Items	Ash	NDF	ADF	CP	EE	Xylan	Mannan
Content (%)	95.04	62.9	38.75	15.61	5.72	13.91	28.78

**Table 5:** Optimal proportion of dual enzymatic hydrolysis.

Items	Xylanase Addition (g)	Mannanase Addition (g)	RS Content (mg/g)	NDF (%)
1	0	0	6.22 ^d^	68.80
2	0.1	0.1	89.49 ^abc^	55.36
3	0.1	0.5	102.88 ^abc^	53.42
4	0.1	1.0	131.98 ^a^	50.09
5	0.5	0.1	89.07 ^abc^	56.40
6	0.5	0.5	94.42 ^abc^	53.10
7	0.5	1.0	124.10 ^ab^	53.58
8	1.0	0.1	51.91 ^c^	61.13
9	1.0	0.5	87.48 ^c^	52.35
10	1.0	1.0	83.43 ^bc^	56.06
SEM			36.37	5.11
*p* value			<0.05	0.06

Note: ^a, b, c, d^ Means in the same column without common superscripts differ significantly (*p* < 0.05).

**Table 6:** Optimization of bacterial ratio.

Items	CP	RS (mg/g)	NDF (%)	pH Value	Total Acid (g/kg)	Viable Count (CFU/mL)
Before Fermentation	21.65 ^b^	14.06 ^c^	55.83 ^a^	5.82 ^b^	1.04 ^d^	1.9 × 10^9 a^
Control Group	21.60 ^b^	9.26 ^d^	55.70 ^a^	6.02 ^a^	0.98 ^d^	0
Group 1 (5:5)	22.28 ^b^	22.48 ^b^	51.33 ^b^	5.30 ^c^	1.53 ^c^	4.9 × 10^4 c^
Group 2 (7:3)	23.29 ^a^	49.37 ^a^	51.06 ^b^	4.34 ^d^	3.15 ^a^	5.6 × 10^5 b^
Group 3 (3:7)	22.14 ^b^	22.50 ^b^	51.81 ^b^	4.33 ^d^	2.44 ^b^	5.0 × 10^4 c^
SEM	0.76	14.68	2.29	0.73	0.89	
*p*-value	<0.05	<0.01	<0.05	<0.05	<0.01	<0.01

Note: ^a, b, c, d^ Means in the same column without common superscripts differ significantly (*p* < 0.05).

**Table 7:** Optimization of inoculation amount.

Items	CP	RS (mg/g)	NDF (%)	pH Value	Total Acid (g/kg)	Viable Count (CFU/mL)
Before Fermentation	23.30 ^c^	13.98 ^d^	58.48	5.82 ^b^	1.43 ^c^	2.1×10^8 a^
Control Group	21.60 ^d^	9.26 ^e^	55.72	6.01 ^a^	0.99 ^d^	0
Group 1 (5%)	24.65 ^b^	30.73 ^a^	50.94	4.62 ^c^	2.79 ^b^	1.1×10^6 b^
Group 2 (10%)	25.50 ^a^	28.97 ^b^	53.09	4.59 ^c^	3.15 ^a^	3.2×10^5 c^
Group 3 (15%)	25.76 ^a^	25.10 ^c^	56.13	4.53 ^c^	3.15 ^a^	3.5×10^5 c^
SEM	1.69	8.59	3.12	0.65	0.96	
*p*-value	<0.05	<0.01	0.12	<0.05	<0.01	<0.01

Note: ^a, b, c, d, e^ Means in the same column without common superscripts differ significantly (*p* < 0.05).

**Table 8:** Optimization of water content.

Items	CP	RS (mg/g)	NDF (%)	pH Value	Total Acid (g/kg)	Viable Count (CFU/mL)
Control Group	21.6 ^f^	9.26 ^c^	55.72 ^e^	6.01 ^a^	0.99 ^e^	0
20% Before Fermentation	22.26 ^e^	14.16 ^c^	60.23 ^c^	5.89 ^b^	1.44 ^c^	2.7 × 10^8 a^
28% Before Fermentation	22.47 ^ce^	23.04 ^b^	61.68 ^b^	5.88 ^b^	1.17 ^d^	2.7 × 10^8 a^
36% Before Fermentation	22.93 ^abc^	22.51 ^b^	63.13 ^a^	5.89 ^b^	0.81 ^e^	2.7 × 10^8 a^
Group 1 (20%)	22.84 ^bce^	32.66 ^a^	50.64 ^f^	4.50 ^d^	2.23 ^b^	2.0 × 10^6 b^
Group 2 (28%)	23.01 ^ab^	26.78 ^a^	59.67 ^d^	4.69 ^c^	2.51 ^a^	1.6 × 10^5 c^
Group 3 (36%)	23.12 ^a^	24.83 ^b^	60.19 ^c^	4.45 ^d^	2.38 ^b^	1.8 × 10^5 c^
SEM	0.51	8.05	4.07	0.62	2.05	
*p*-value	<0.01	<0.01	<0.01	<0.01	<0.01	<0.01

Note: ^a, b, c, d, e, f^ Means in the same column without common superscripts differ significantly (*p* < 0.05).

**Table 9:** Optimization of fermentation time.

Items	CP	RS (mg/g)	NDF (%)	pH Value	Total Acid (g/kg)	Viable Count (CFU/mL)
Before Fermentation	22.26	14.16 ^b^	60.23 ^a^	5.89 ^a^	1.44 ^d^	2.7 × 10^8 a^
Group 1 (3 d)	22.81	42.33 ^a^	50.64 ^b^	5.28 ^c^	1.62 ^b^	5.4 × 10^6 b^
Group 2 (5 d)	22.84	32.66 ^a^	58.56 ^a^	5.53 ^b^	1.53 ^c^	2.6 × 10^5 c^
Group 3 (7 d)	22.81	35.68 ^a^	58.22 ^a^	5.13 ^d^	2.34 ^a^	1.8 × 10^5 c^
SEM	0.31	10.49	4.01	0.28	0.38	
*p*-value	0.19	<0.01	<0.01	<0.05	<0.01	<0.01

Note: ^a, b, c, d^ Means in the same column without common superscripts differ significantly (*p* < 0.05).

**Table 10:** Effects of pretreated palm kernel cake on growth performance in broilers (n = 5).

Items	CON	PKC1	PKC2	EPKC1	EPKC2	FEPKC1	FEPKC2	SEM	*p* Value
d 1~21									
ADG, g/d	23.40 ^c^	22.24 ^c^	26.04 ^b^	27.22 ^b^	27.88 ^b^	29.06 ^a^	27.51 ^b^	2.46	<0.01
ADFI, g/d	31.90 ^c^	33.24 ^b^	35.92 ^a^	33.18 ^b^	35.31 ^a^	35.38 ^a^	33.29 ^b^	2.06	<0.01
F:G	1.36 ^b^	1.49 ^a^	1.38 ^b^	1.22 ^d^	1.27 ^c^	1.22 ^d^	1.21 ^d^	0.13	<0.01
d 21~42									
ADG, g/d	62.28 ^bc^	59.99 ^cd^	57.77 ^d^	60.23 ^cd^	59.81 ^cd^	65.44 ^a^	63.11 ^ab^	2.95	<0.01
ADFI, g/d	91.56 ^c^	97.70 ^b^	108.11 ^a^	96.09 ^bc^	104.81 ^a^	98.78 ^b^	106.75 ^a^	6.74	<0.01
F:G	1.47 ^e^	1.63 ^cd^	1.87 ^a^	1.59 ^d^	1.75 ^b^	1.51 ^e^	1.69 ^bc^	0.14	<0.01
d 1~42									
ADG, g/d	42.89 ^c^	43.12 ^bc^	42.47 ^c^	45.21 ^b^	43.80 ^bc^	47.57 ^a^	45.30 ^b^	2.20	<0.01
ADFI, g/d	61.73 ^c^	65.47 ^b^	72.02 ^a^	64.64 ^bc^	70.06 ^a^	65.44 ^b^	71.06 ^a^	4.18	<0.01
F:G	1.44 ^d^	1.52 ^c^	1.70 ^a^	1.43 ^d^	1.60 ^b^	1.38 ^d^	1.57 ^bc^	0.11	<0.01

Note: ^a, b, c, d, e^ Means in the same column without common superscripts differ significantly (*p* < 0.05).

**Table 11:** Effects of pretreated palm kernel cake on nutrient digestibility in broilers (%, n = 5).

Items	CON	PKC1	PKC2	EPKC1	EPKC2	FEPKC1	FEPKC2	SEM	*p* Value
d 21									
DM	66.00 ^bc^	64.88 ^c^	64.09 ^c^	69.25 ^ab^	62.33 ^cd^	70.70 ^a^	60.12 ^d^	4.19	<0.01
CP	62.22 ^ab^	57.57 ^bc^	53.79 ^cd^	58.03 ^bc^	53.53 ^cd^	66.95 ^a^	57.39 ^d^	6.17	<0.01
EE	87.21	87.98	91.62	92.18	91.57	90.87	91.67	3.35	0.05
NDF	23.57	18.66	21.60	24.22	19.98	21.05	18.32	5.79	0.34
ADF	8.24 ^b^	11.56 ^b^	19.14 ^a^	17.25 ^a^	9.37 ^b^	11.36 ^b^	8.04 ^b^	4.68	<0.01
d 42									
DM	76.55 ^a^	67.71 ^bc^	63.24 ^d^	67.41 ^c^	63.34 ^d^	70.25 ^b^	64.23 ^d^	4.83	<0.01
CP	63.91 ^a^	56.46 ^b^	46.46 ^c^	54.27 ^b^	45.14 ^c^	57.22 ^b^	45.94 ^c^	7.55	<0.01
EE	87.75 ^b^	92.7 ^a^	92.58 ^a^	92.14 ^a^	93.14 ^a^	91.99 ^a^	89.87 ^ab^	2.87	0.01
NDF	19.84	16.72	24.81	21.01	23.15	24.47	18.04	5.59	0.17
ADF	19.17 ^ab^	12.51 ^bc^	11.79 ^bc^	14.74 ^ab^	14.84 ^ab^	21.07 ^a^	6.08 ^c^	6.33	<0.01

Note: ^a, b, c, d^ Means in the same column without common superscripts differ significantly (*p* < 0.05).

**Table 12:** Effects of pretreated palm kernel cake on serum antioxidant indices of broilers (n = 5).

Items	CON	PKC1	PKC2	EPKC1	EPKC2	FEPKC1	FEPKC2	SEM	*p* Value
21 d									
T-SOD, U/mL	135.22 ^c^	244.48 ^ab^	228.91 ^b^	253.69 ^ab^	281.81 ^a^	239.24 ^b^	236.53 ^b^	52.50	<0.01
MDA, nmol/mL	3.63 ^a^	2.95 ^abc^	3.47 ^ab^	2.64 ^c^	2.31 ^c^	2.75 ^bc^	2.89 ^abc^	0.69	0.01
T-AOC, pg/mL	0.55	0.52	0.60	0.52	0.59	0.55	0.57	25.60	0.57
42 d									
T-SOD, U/mL	125.16 ^b^	249.40 ^a^	253.57 ^a^	268.31 ^a^	262.27 ^a^	261.31 ^a^	263.50 ^a^	56.40	<0.01
MDA, nmol/mL	3.66 ^a^	2.79 ^ab^	3.17 ^ab^	2.41 ^b^	2.73 ^b^	2.66 ^b^	2.87 ^ab^	0.70	<0.01
T-AOC, pg/mL	0.49	0.42	0.41	0.43	0.45	0.46	0.49	0.06	0.18

Note: ^a, b, c^ Means in the same column without common superscripts differ significantly (*p* < 0.05).

**Table 13:** Effects of pretreated palm kernel cake on serum immune indices of broilers (n = 5).

Items	CON	PKC1	PKC2	EPKC1	EPKC2	FEPKC1	FEPKC2	SEM	*p* Value
21 d									
IgA, µg/mL	1.06 ^bc^	1.01 ^bc^	0.83 ^c^	1.21 ^bc^	1.22 ^b^	1.31 ^b^	1.72 ^a^	0.36	<0.01
IgG, mg/mL	6.36 ^a^	5.90 ^ab^	4.48 ^c^	5.86 ^ab^	5.03 ^bc^	5.97 ^ab^	6.60 ^a^	1.03	<0.01
IgM, µg/mL	1.15 ^a^	0.72 ^b^	0.49 ^c^	0.72 ^b^	0.67 ^bc^	0.74 ^b^	0.85 ^b^	0.24	<0.01
42 d									
IgA, µg/mL	0.99 ^b^	1.90 ^a^	1.84 ^a^	1.96 ^a^	2.00 ^a^	1.82 ^a^	2.13 ^a^	0.44	<0.01
IgG, mg/mL	6.60 ^b^	7.73 ^a^	7.26 ^ab^	7.50 ^ab^	8.08 ^a^	7.05 ^ab^	7.47 ^ab^	0.80	0.05
IgM, µg/mL	1.32 ^a^	0.88 ^b^	0.92 ^b^	0.98 ^b^	1.01 ^b^	0.86 ^b^	0.95 ^b^	0.19	<0.01

Note: ^a, b, c^ Means in the same column without common superscripts differ significantly (*p* < 0.05).

**Table 14:** Effects of pretreated palm kernel cake on immune organ indicese of broilers (Unit: g/kg, n = 5).

Items	CON	PKC1	PKC2	EPKC1	EPKC2	FEPKC1	FEPKC2	SEM	*p* Value
d 21									
Thymus index	2.50	1.75	1.90	1.57	1.93	2.40	2.33	0.76	0.34
Spleen index	1.16	0.78	0.88	0.73	0.86	0.93	0.97	0.32	0.37
Bursae index of Fabricius	3.00	2.26	2.60	2.13	2.82	2.32	2.79	0.64	0.21
d 42									
Thymus index	2.05	2.27	2.17	2.35	2.01	1.89	2.23	0.61	0.91
Spleen index	1.04	1.01	1.05	0.94	1.25	1.00	0.92	0.25	0.61
Bursae index of Fabricius	2.60	2.01	1.85	1.84	1.70	2.24	2.05	0.70	0.42

**Table 15:** Effects of pretreated palm kernel cake on intestinal tissue morphology of broilers (n = 5).

Items	CON	PKC1	PKC2	EPKC1	EPKC2	FEPKC1	FEPKC2	SEM	*p* Value
d 21									
Jejunum									
Villus height, μm	810.98	796.15	945.80	917.74	878.71	929.22	725.71	216.01	0.81
Crypt depth, μm	145.89	157.03	146.52	146.79	169.56	168.59	108.94	28.00	0.06
Villus height/Crypt depth	5.54	5.10	6.28	6.25	4.82	5.75	6.66	1.28	0.35
Ileum									
Villus height, μm	556.82	573.93	606.01	663.24	569.17	606.53	658.73	107.11	0.69
Crypt depth, μm	164.20	136.22	138.58	138.54	142.56	125.34	156.62	19.03	0.05
Villus height/Crypt depth	3.46	4.26	4.37	4.80	4.00	4.94	4.18	0.85	0.22
d 42									
Jejunum									
Villus height, μm	1334.22	1036.57	1370.42	1283.49	1303.73	1323.08	1334.38	202.61	0.18
Crypt depth, μm	203.35	187.97	171.37	211.13	152.41	164.14	166.34	37.78	0.17
Villus height/Crypt depth	6.58 ^c^	5.53 ^d^	7.99 ^b^	6.51 ^c^	8.81 ^a^	8.02 ^b^	8.08 ^b^	1.63	0.01
Ileum									
Villus height, μm	828.66	709.62	863.60	917.25	1025.38	771.22	1022.86	221.36	0.25
Crypt depth, μm	198.31 ^a^	144.27 ^b^	125.40 ^b^	158.14 ^b^	145.45 ^b^	148.87 ^b^	150.73 ^b^	34.12	0.02
Villus height/Crypt depth	4.41 ^b^	4.93 ^ab^	6.83 ^a^	6.31 ^ab^	6.88 ^a^	5.16 ^ab^	6.73 ^a^	1.51	0.03

Note: ^a, b, c, d^ Means in the same column without common superscripts differ significantly (*p* < 0.05).

## Data Availability

The data presented in this study will be made available upon request from the authors.

## References

[1] Alshelmani M.I., Kaka U., Abdalla E.A., Humam A.M., Zamani H.U. (2021). Effect of feeding fermented and non-fermented palm kernel cake on the performance of broiler chickens: A review. World’s Poult. Sci. J..

[2] Nahrowi N., Saidah N., Jayanegara A., Sofyan A. (2024). Physicochemical properties of palm kernel cake processed by combination of screening and blowing technology. AIP Conf. Proc..

[3] Huang H., Lin X., Meng X., Liu Y., Fan J., Zhu L., Chen J., Zhang L., Mi H., Deng J. (2024). Effects of replacing wheat bran with palm kernel cake or fermented palm kernel cake on the growth performance, intestinal microbiota and intestinal health of tilapia (GIFT, *Oreochromis niloticus*). Front. Nutr..

[4] Akpanabiatu M., Ekpa O., Mauro A., Rizzo R. (2001). Nutrient composition of Nigerian palm kernel from the dura and tenera varieties of the oil palm (*Elaeis guineensis*). Food Chem..

[5] Onifade A.A., Babatunde G.M. (1998). Comparison of the utilisation of palm kernel meal, brewers’ dried grains and maize offal by broiler chicks. Br. Poult. Sci..

[6] Keong N.W. (2005). Researching the Use of Palm Kernel Cake in Aquaculture Feeds. Palm Oil Dev..

[7] Mustafa M.F., Alimon A.R., Zahari M.W., Idris I., Bejo M.H. (2003). Nutrient Digestibility of Palm Kernel Cake for Muscovy Ducks. Asian-Australas. J. Anim. Sci..

[8] Sue T.T. (2001). Quality and Characteristics of Malaysian Palm Kernel Cakes/Expellers. Palm Oil Dev..

[9] Zulkifli I., Rahayu H.S.I., Alimon A.R. (2009). Gut microflora and intestinal morphology of commercial broiler chickens and red jungle fowl fed diets containing palm kernel meal. Archiv. Geflugelkd..

[10] Zubaidah S., Ariyadi B., Hanim C., Baskara A.P. (2024). Performance of broiler chickens fed palm kernel cake with enzyme supplementation. IOP Conference Series: Earth and Environmental Science.

[11] Turner M., Saville B. (2022). Technoeconomic evaluation of protein-rich animal feed and ethanol production from palm kernel cake. Biofuels Bioprod. Biorefining.

[12] Soares C., Rossa F., da Silva F.F., da Silva A.P.G., Santos L.V., Júnior D.M.d.L., Silva R.R. (2024). Effect of palm kernel cake inclusion in the supplement of pasture-finished heifers on performance, carcass traits, and meat quality. N. Z. J. Agric. Res..

[13] Saminathan M., Mohamed W.N., Noh A.M., Ibrahim N.A., Fuat M.A., Dian N.L., Ramiah S.K. (2020). Potential of feeding crude palm oil and co-products of palm oil milling on laying hens’ performance and egg quality: A review. J. Oil Palm Res..

[14] Kango N., Jana U.K., Choukade R., Nath S. (2022). Advances in prebiotic mannooligosaccharides. Curr. Opin. Food Sci..

[15] Ong W.L., Li Z., Ng K.-H., Zhou K. (2024). Improving Mannanase Production in Bacillus subtilis for Fibre Hydrolysis during Solid-State Fermentation of Palm Kernel Meal. Biochem. Eng. J..

[16] Dong W., Dong S., Li Y., Lei Y., Peng N., Liang Y., Zhao S., Ge X. (2022). Comprehensive utilization of palm kernel cake for producing mannose and manno-oligosaccharide mixture and yeast culture. Appl. Microbiol. Biotechnol..

[17] Khubber S., Marti-Quijal F.J., Tomasevic I., Remize F., Barba F.J. (2022). Lactic acid fermentation as a useful strategy to recover antimicrobial and antioxidant compounds from food and by-products. Curr. Opin. Food Sci..

[18] Cao Y., Xu M., Lu J., Cai G. (2024). Simultaneous microbial fermentation and enzymolysis: A biotechnology strategy to improve the nutritional and functional quality of soybean meal. Food Rev. Int..

[19] (2018). Determination of Activity of β-Mannanase as Feed Additive—Spectrophotometric Method.

[20] (2006). Determination of Neutral Detergent Fiber in Feedstuffs.

[21] (1994). Method for the Determination of Crude Protein in Feedstuffs.

[22] (2006). Determination of Moisture and Other Volatile Mater Content in Feeds.

[23] (2007). Determination of Acid Detergent Fiber in Feedstuff (ADF).

[24] Rutherfurd S.M. (2009). and G.S. Gilani, Amino Acid Analysis. Curr. Protoc. Protein Sci..

[25] Wang W., Zhang C., Zhao Y., Wang L., Tang M., Yin Y., Xiong X., Yin Y., Kim S.W., Tang X. (2024). Methodology to Study Tryptophan Metabolism. Tryptophan in Animal Nutrition and Human Health.

[26] (2021). National Food Safety Standard—Determination of Total Acid in Foods.

[27] (2020). Formula Feeds for Layers and Broilers.

[28] (2006). Determination of Chromium in Feeds.

[29] Oladokun A.A., Rahman W.A., Suparjo N.M. (2016). Prospect of maximising palm kernel cake utilization for livestock and poultry in Malaysia: A review. J. Biol. Agric. Healthc..

[30] Sathitkowitchai W., Ayimbila F., Nitisinprasert S., Keawsompong S. (2022). Selection of pretreatment method and mannanase enzyme to improve the functionality of palm kernel cake. J. Biosci. Bioeng..

[31] Sundu B., Asril A., Hafsah H., Saloko F. (2024). Enzymatic hydrolyzation of fermented palm kernel meal with the addition of ammonium sulfate in poultry diet. IOP Conference Series: Earth and Environmental Science.

[32] Malgas S., MMafa S., Pletschke B.I. (2020). The effects of xylanase synergistic interactions during lignocellulose degradation and their significance for industry. Industrial Applications of Glycoside Hydrolases.

[33] Tenkanen M., Makkonen M., Perttula M., Viikari L., Teleman A. (1997). Action of Trichoderma reesei mannanase on galactoglucomannan in pine kraft pul. J. Biotechnol..

[34] Zhang W., Bao C., Wang J., Zang J., Cao Y. (2020). Administration of Saccharomyces boulardii mafic-1701 improves feed conversion ratio, promotes antioxidant capacity, alleviates intestinal inflammation and modulates gut microbiota in weaned piglets. J. Anim. Sci. Biotechnol..

[35] Zhang D., Tan B., Zhang Y., Ye Y., Gao K. (2022). Improved nutritional and antioxidant properties of hulless barley following solid-state fermentation with *Saccharomyces cerevisiae* and *Lactobacillus plantarum*. J. Food Process. Preserv..

[36] Wu H., Liu H.-N., Ma A.-M., Zhou J.-Z., Xia X.-D. (2022). Synergetic effects of *Lactobacillus plantarum* and *Rhizopus oryzae* on physicochemical, nutritional and antioxidant properties of whole-grain oats (*Avena sativa* L.) during solid-state fermentation. LWT.

[37] Wang Z., Song S., Liu J., Bai X., Ye G., Liu J. (2024). Solid-state fermentation by *Aspergillus niger* and *Lactobacillus plantarum* improved the nutritional and physicochemical properties of wheat bran and whole wheat bread. Int. J. Food Sci. Technol..

[38] Chen L., Zhao Z., Yu W., Zheng L., Li L., Gu W., Xu H., Wei B., Yan X. (2021). Nutritional quality improvement of soybean meal by *Bacillus velezensis* and Lactobacillus plantarum during two-stage solid-state fermentation. AMB Express.

[39] Zhang D., Tan B. (2021). Effects of different solid-state fermentation ratios of *S. cerevisiae* and *L. plantarum* on physico-chemical properties of wheat bran and the quality of whole wheat bread. J. Sci. Food Agric..

[40] Haokok C., Lunprom S., Reungsang A., Salakkam A. (2023). Efficient production of lactic acid from cellulose and xylan in sugarcane bagasse by newly isolated *Lactiplantibacillus plantarum* and *Levilactobacillus brevis* through simultaneous saccharification and co-fermentation process. Heliyon.

[41] Jana U.K., Suryawanshi R.K., Prajapati B.P., Kango N. (2021). Prebiotic mannooligosaccharides: Synthesis, characterization and bioactive properties. Food Chem..

[42] Muthusamy K., Soundharrajan I., Srisesharam S., Kim D., Kuppusamy P., Lee K.D., Choi K.C. (2020). Probiotic characteristics and antifungal activity of Lactobacillus plantarum and its impact on fermentation of Italian ryegrass at low moisture. Appl. Sci..

[43] Morales E.M., Zajul M., Goldman M., Zorn H., Angelis D.F. (2020). Effects of solid-state fermentation and the potential use of cassava by-products as fermented food. Waste Biomass Valorization.

[44] Wason S., Verma T., Subbiah J. (2021). Validation of process technologies for enhancing the safety of low-moisture foods: A review. Compr. Rev. Food Sci. Food Saf..

[45] Lau S.Y.L., Halim M.A., Telajan E.R.A.D., Wong C.M.V.L. (2022). Emericella nidulans (4DP5), Cladosporium herbarum (7DF12) and Bacillus subtilis improve the nutritional value of palm kernel cake (PKC) through solid-state fermentation (SSF). Malays. J. Microbiol..

[46] Azizi M.N., Loh T.C., Foo H.L., Teik Chung E.L. (2021). Is palm kernel cake a suitable alternative feed ingredient for poultry?. Animals.

[47] Anyanwu N.J., Obilonu B.C., Odoemelam V.U., Etela I., Kalio G.A., Ekpe I.I. (2020). Growth performance and haematological characteristics of broiler finisher chickens fed palm kernel cake as partial replacement for maize and Soya bean. Niger. J. Anim. Prod..

[48] Koranteng A.A.-A., Gbogbo K.A., Adjei-Mensah B., Bouassi T., Agbehadzi R.K., Tona K. (2023). Influence of palm kernel cake on the growth performance, gut health and hematochemical indices of slow-growing broilers. J. Appl. Anim. Res..

[49] Egbune E.O., Tonukari N.J. (2023). Fermented mixture of cassava roots and palm kernel cake can substitute for maize in poultry feed formulation. Afr. J. Biochem. Res..

[50] Adli D.N., Sjofjan O., Natsir M.H., Nuningtyas Y.F., Sholikah N.U., Marbun A.C. (2020). The effect of replacing maize with fermented palm kernel meal (FPKM) on broiler performance. Livest. Res. Rural Dev..

[51] Hakim A.H., Zulkifli I., Farjam A.S., Awad E.A. (2021). Feeding fermented palm kernel cake with higher levels of dietary fat improved gut bacterial population and blood lipid concentration but not the growth performance in broiler chickens. Ital. J. Anim. Sci..

[52] Hakim A.H., Zulkifli I., Farjam A.S., Awad E.A., Ramiah S.K. (2022). Impact of feeding fermented palm kernel cake and high dietary fat on nutrient digestibility, enzyme activity, intestinal morphology and intestinal nutrient transporters mRNA expression in broiler chickens under hot and humid conditions. Animals.

[53] Alshelmani M.I., Loh T.C., Foo H.L., Sazili A.Q., Lau W.H. (2016). Effect of feeding different levels of palm kernel cake fermented by Paenibacillus polymyxa ATCC 842 on broiler growth performance, blood biochemistry, carcass characteristics, and meat quality. Anim. Prod. Sci..

[54] Walugembe M., Hsieh J.C.F., Koszewski N.J., Lamont S.J., Persia M.E., Rothschild M.F. (2015). Effects of dietary fiber on cecal short-chain fatty acid and cecal microbiota of broiler and laying-hen chicks. Poult. Sci..

[55] Zhou M., Tao Y., Lai C., Huang C., Yong Q. (2021). Dietary mannanoligosaccharide supplementation improves growth performance, intestinal integrity, serum immunity, and antioxidant capacity of partridge shank chickens. J. Poult. Sci..

[56] Yu E., Chen D., Yu B., Luo Y., Zheng P., Yin H., Mao X., Huang Z., Yu J., Luo J. (2021). Amelioration of enterotoxigenic Escherichia coli-induced disruption of intestinal epithelium by manno-oligosaccharide in weaned pigs. J. Funct. Foods.

[57] Yousaf S., Nouman H.M., Ahmed I., Husain S., Waseem M., Nadeem S., Tariq M., Sizmaz O., Chudhry M.F. (2022). A Review of Probiotic Applications in Poultry: Improving Immunity and Having Beneficial Effects on Production and Health. Adv. Microbiol. Sciendo.

[58] Zhang J., Yang Y., Han H., Zhang L., Wang T. (2021). Bisdemethoxycurcumin attenuates lipopolysaccharide-induced intestinal damage through improving barrier integrity, suppressing inflammation, and modulating gut microbiota in broilers. J. Anim. Sci..

